# Antipsychotic Drug Trifluoperazine Suppresses Colorectal Cancer by Inducing G0/G1 Arrest and Apoptosis

**DOI:** 10.3389/fphar.2019.01029

**Published:** 2019-09-13

**Authors:** Yong Xia, Chengsen Jia, Qiang Xue, Jinrui Jiang, Yao Xie, Ranran Wang, Zhiqiang Ran, Fuyan Xu, Yiwen Zhang, Tinghong Ye

**Affiliations:** ^1^Department of Rehabilitation Medicine and Laboratory of Liver Surgery, State Key Laboratory of Biotherapy and Cancer Center, West China Hospital, Sichuan University and Collaborative Innovation Center for Biotherapy, Chengdu, China; ^2^Key Laboratory of Rehabilitation Medicine, West China Hospital, Sichuan University, Chengdu, China; ^3^West China School of Pharmacy, Sichuan University, Chengdu, China; ^4^Department of Gynecology and Obstetrics, Sichuan Academy of Medical Sciences & Sichuan Provincial People’s Hospital, Chengdu, China

**Keywords:** trifluoperazine hydrochloride, colorectal cancer, cell cycle arrest, apoptosis, programmed death-1 ligand 1 (PD-L1)

## Abstract

Repurposing existing drugs for cancer treatment is an effective strategy. An approved antipsychotic drug, trifluoperazine (TFP), has been reported to have potential anticancer effects against several cancer types. Here, we investigated the effect and molecular mechanism of TFP in colorectal cancer (CRC). *In vitro* studies showed that TFP induced G0/G1 cell cycle arrest to dramatically inhibit CRC cell proliferation through downregulating cyclin-dependent kinase (CDK) 2, CDK4, cyclin D1, and cyclin E and upregulating p27. TFP also induced apoptosis, decreased mitochondrial membrane potential, and increased reactive oxygen species levels in CRC cells, indicating that TFP induced mitochondria-mediated intrinsic apoptosis. Importantly, TFP significantly suppressed tumor growth in two CRC subcutaneous tumor models without side effects. Interestingly, TFP treatment increased the expression levels of programmed death-1 ligand 1 (PD-L1) in CRC cells and programmed death-1 (PD-1) in tumor-infiltrating CD4+ and CD8+ T cells, implying that the combination of TFP with an immune checkpoint inhibitor, such as an anti-PD-L1 or anti-PD-1 antibody, might have synergistic anticancer effects. Taken together, our study signifies that TFP is a novel treatment strategy for CRC and indicates the potential for using the combination treatment of TFP and immune checkpoint blockade to increase antitumor efficiency.

## Introduction

Colorectal cancer (CRC) is one of the leading causes of cancer-related mortalities in the world, with ∼830,000 deaths per year (Bray et al., 2018). The main therapeutic options for CRC are surgery, chemotherapy, targeted therapy, and radiotherapy. Despite advances in therapeutic strategies for CRC, the 5-year survival rate of CRC patients remains poor because of high recurrence and metastasis incidence (Wang and Li, 2012). Therefore, it is necessary to explore new therapeutic drugs or combinational treatments for CRC.

Despite the tremendous resources invested, the process of anticancer research and development is increasingly challenging due to high failure rates and withdrawal risks (Gupta et al., 2013; Basso et al., 2018). Drug repurposing, alternatively called “new uses for old drugs,” has attracted considerable attention from both academic institutions and pharmaceutical companies (Turanli et al., 2018). The main advantage of this strategy is that the pharmacodynamics, pharmacokinetics, and safety profiles of the drugs are well known, which signiﬁcantly reduces the time spent on the development process and accelerates their new application to other diseases (Gupta et al., 2013; Dilly et al., 2017). There are many drug repurposing successes in cancer treatment—for example, rapamycin, an immunosuppressant, was repurposed for treating CRC, lymphoma, and leukemia, and zoledronic acid, an anti-bone resorption medicine, was repurposed for treating the bone metastasis of breast cancer and lung cancer (Gupta et al., 2013; Dilly et al., 2017).

Schizophrenic patients prescribed neuroleptic drugs have been shown to have a lower frequency of cancer, implying that these drugs have potential anticancer abilities (Dalton et al., 2006). Indeed, some anti-schizophrenic drugs, including chlorpromazine and aripiprazole, have exhibited anticancer effects in preclinical studies (Oliva et al., 2017; Mi et al., 2018). These findings demonstrate that anti-schizophrenic agents have overlooked anticancer potential.

Trifluoperazine (TFP) is a commonly prescribed antipsychotic drug approved in 2001. It exerts antipsychotic effects by modulating dopaminergic signaling (Marques et al., 2004). Previous studies have reported its beneficial treatment effects for some cancers in preclinical studies either alone or combined with other anticancer agents, such as triple negative breast cancer, glioblastoma, and lung cancer (Chi-Tai et al., 2012; Kang et al., 2017; Fancy et al., 2018). It could overcome EGFR inhibitor and chemotherapy resistance of lung cancer by targeting cancer stem-like cells. It also showed efficacies in suppressing metastasis in several cancers (Ashleigh et al., 2015; Feng et al., 2018; Liu et al., 2018; Goyette et al., 2019). TFP induced apoptosis of cancer cells and disrupting cell cycle progression *in vitro* and *in vivo* in numerous models. It exhibits antitumor effects by regulating different signaling pathways. Dopaminergic signaling is involved in the anticancer abilities of TFP in treating breast cancer (Liu et al., 2018). TFP is known to be a calmodulin inhibitor. One of other proposed modes of effects of TFP is its ability of binding to a well-known Ca^2+^ binding protein, calmodulin (CaM) (Kang et al., 2017; Park et al., 2019). TFP is also shown to promote FOXO3 nuclear localization and activation to suppress breast cancer (Park et al., 2016). In hepatocellular carcinoma, TFP could activate forkhead box O1 (FOXO1)–related signals to inhibit tumor growth (Jiang et al., 2017). The receptor tyrosine kinase AXL is another target of TFP to reduce growth and metastasis of breast cancer (Goyette et al., 2019). Immune-based cancer therapy is a promising strategy to treat cancer. Interestingly, TFP was found to modulate immunologic parameters. It reduced lymphocyte proliferation both *in vitro* and *in vivo* and might cause immunosuppression (Roudebush et al., 1991). During sepsis, it reduced inflammatory response by inhibiting cytokine release in LPS-stimulated macrophages and dendritic cells (Park et al., 2019). The above information indicated that immune system might be evolved in TFP’s anticancer effects.

However, whether TFP could inhibit CRC and the underlying mechanism remains unknown. In this study, we found that TFP significantly reduced the growth of several CRC cell lines *in vitro* and suppressed the growth of subcutaneous tumors of both human and mouse CRC without causing obvious side effects *in vivo*. Mechanistically, TFP induced G0/G1 cell cycle arrest and mitochondria-mediated intrinsic apoptosis. Strikingly, programmed death-1 ligand 1 (PD-L1) expression levels in tumor cells and programmed death-1 (PD-1) expression levels in tumor-infiltrating CD4+ and CD8+ T cells were increased after TFP treatment, implying that the combination of TFP with an anti-PD-L1 or anti-PD-1 antibody might have stronger synergistic anticancer effects. In sum, this is the first study to indicate that repurposing TFP is a novel and effective treatment strategy for CRC.

## Materials and Methods

### Materials

MTT (3-[4,5-dimethyl-2-thiazolyl]-2,5-diphenyl-2-H-tetrazolium bromide, thiazolyl blue tetrazolium bromide), propidium iodide (PI), rhodamine-123 (Rh123), N-acetyl-L-cysteine (NAC), and Hoechst 33342 were purchased from Sigma (St. Louis, MO). TFP, 5-fluorouracil (5-FU) and oxaliplatin were purchased from Innochem (Beijing, China). Z-LE(OMe)HD(OMe)-FMK (#KGA8261) was purchased from Nanjing KeyGen Biotech (Nanjing, China). For all *in vitro* assays, TFP was dissolved in DMSO as a 20 mM stock solution. It is dissolved in DMSO/Cremophor EL/saline at 2.5:12.5:85 v/v for the *in vivo* experiments. Antibodies against caspase-3 (#9664s), cyclin-dependent kinase (CDK) 2 (#2546), cyclin D1 (#2978), P27 (#3688), AKT (#4658s), p-AKT (#4060s), NF-κB P65 (#8242), and p-NF-κB P65 (#3033) were purchased from Cell Signaling Technology. Antibodies against Bax (#610982), Bcl-2 (#2610538), cyclin E (#51-14596R), mouse PD-L1 (#558091), and mouse PD-1 (#562671) were purchased from BD Bioscience. Antibodies against β-actin (#200068-8F10), and CDK4 (#200540) were purchased from Zen Bioscience. Antibodies against human PD-L1 (#329707), mouse CD45 (#103112), mouse CD4 (#100408), and mouse CD8 (#100706) were purchased from BioLegend. Secondary antibodies were purchased from Zhongshan Jinqiao Biotechnology Group.

### Cell Lines and Cell Culture

Human CRC cell line SW620, HCT116, mouse CRC cell line CT26, normal human colon epithelial cell line HCoEpiC, and mouse embryo fibroblast cell line NIH-3T3 were purchased from the American Type Culture Collection (ATCC) within the past 5 years. The cells were cultured in DME/F-12 medium supplemented with 10% FBS, penicillin (100 U/ml), and streptomycin (0.1 mg/ml) in a humidiﬁed incubator with 5% CO_2_ at 37°C.

### Cell Viability Assay and Colony Formation Assay

MTT was used to assess CRC cell viability according to our previous study (Xia et al., 2014a). Cells were seeded on 96-well plates at 1,500 to 3,000 cells/well/100 µl and allowed to attach for 24 h. Then, 100 µl of medium containing indicated concentrations of TFP, 5-FU, oxaliplatin, and their combinations were added to each well (this time point is defined as 0 h). After 24 and 48 h, 20 µl of MTT solution (5 mg/ml in saline) were added into each well and incubated for 2 to 3 h. After removing the medium, 150 µl of DMSO were added, and the absorbance at 570 nm was measured with a Spectra Max M5 Microplate Spectrophotometer (Molecular Devices). The cell viabilities at 24 and 48 h in each group were normalized to those at 0 h. Blank wasn’t subtracted. The percentages of inhibition were calculated based on the viabilities of vehicle-treated cells. IC_50_ values were calculated using GraphPad Prism 5. Each assay was replicated five times.

Colony formation assays were performed in six-well plates as we described previously (Xia et al., 2014a). Cells were seeded on six-well plates at 800/well and allowed to attach for 24 h. Then, the cells were incubated with indicated concentrations of TFP for 7 to 14 days. After fixing with 4% paraformaldehyde, the cells were stained with crystal violet solution (0.5% in methanol). The percentages of inhibition on colony formation were calculated based on the viabilities of vehicle-treated group. Each assay was replicated three times.

### Calculation of Combination Index (CI)

Combination index (CI) was calculated with free CompuSyn software (Chou, 2006). According to the recommendation of Dr. Dorothy Chou, a CI value below 0.9 indicated synergistic effects of drug combinations. The synergism was further refined as: slight synergism (SS, CI is between 0.85 and 0.9), moderate synergism (MS, CI is between 0.7 and 0.85), and synergism (S, CI is between 0.3 and 0.7) (Chou, 2006; Vujic et al., 2015).

### Cell Cycle and Apoptosis Analysis

CRC cells were plated in 12-well plates and treated with TFP for the indicated time and then stained with buffer containing 50 µg/ml PI and 0.1% Triton X-100 overnight after fixation in 75% ethanol. Cell cycle distribution was measured by flow cytometry (FCM) using ACEA NovoCyte (ACEA Biosciences Inc., Hangzhou, China). Sub-G1 cells were also analyzed. Each assay was replicated three times.

CRC cells were plated in 12-well plates and treated with TFP, NAC (2 mM), Z-LE(OMe)HD(OMe)-FMK (10 μM), and their combinations for the indicated time; then, the percentages of apoptosis were measured as we previously described using an Annexin V-FITC/PI Apoptosis Detection Kit or Annexin V-PE/7-AAD Apoptosis Detection Kit (BD Bioscience) by FCM (Xia et al., 2014a). Each assay was replicated three times.

### Morphological Analysis of Cell Nuclei

After treatment with TFP for 24 h, cells were stained with Hoechst 33342 (10 µg/ml) for 30 min in the dark after washing with PBS and fixing in 4% paraformaldehyde. The morphologies of the nuclei were then examined with an inverted fluorescence microscope (Olympus, Tokyo, Japan). Each assay was replicated three times.

### Detection of Mitochondrial Membrane Potential (Δψm) and Reactive Oxygen Species (ROS) Levels in Cells

Rh123 (5 µg/ml) and DCFH-DA (10 µM) were used to measure ΔΨm and ROS levels, respectively. CRC cells were plated in 12-well plates and treated with TFP for 24 h. Then, the cells were incubated with Rh123 (5 µg/ml) or DCFH-DA (10 µM) for additional 30 min at 37°C in the dark. After washing with PBS, ΔΨm or ROS levels were measure by FCM (Xia et al., 2014a). Each assay was replicated three times.

### Immunoblot Analysis

The cell lysis buffer (Beyotime, Shanghai, China) was supplemented with protease inhibitors Cocktail and PhosSTOP Phosphatase Inhibitors (Roche Diagnostics, UK) before use. Cells were treated with TFP for the indicated time, and then, whole cell lysates were prepared in the above buffer. Protein concentrations were measured using a BCA Protein Assay Kit (Beyotime, Shanghai, China). Then, equal amounts of protein (20–40µg) were resolved by 10% SDS-PAGE. Gels were then transferred onto nitrocellulose (NC) membranes (Millipore, MA, US). After blocking with 5% bovine serum albumin (BSA), the membranes were incubated with the specific primary antibodies at 4°C overnight. After washing with TBST buffer, the membranes were incubated with horseradish peroxidase (HRP)–conjugated secondary antibodies for 2 h at room temperature. Finally, the protein levels were visualized using Immobilon^TM^ Western Chemiluminescent HRP Substrate system (Millipore, MA, US) as we described previously (Xia et al., 2014a). The band intensities from three independent experiments were quantified by ImageJ software (NIH, Bethesda, MD, US).

### Subcutaneous Tumor Models

The animal experiments were approved by the Ethics Committee of Sichuan University. Human CRC HCT116 (1 × 10^7^ cells per mouse) cells were injected subcutaneously into the right flank of 8- to 10-week-old female BALB/c nude mice (HFK Bioscience, Beijing, China). Because of the lack of a thymus, nude mice cannot generate mature T lymphocytes and thus are immunodeficient. Mouse CRC CT26 (1 × 10^6^ cells per mouse) cells were injected subcutaneously into the right flank of immune-competent 8- to 10-week-old female BALB/c mice (HFK Bioscience, Beijing, China). The tumor volumes were calculated according to the following equation: volume (mm^3^) = 0.5 × length (mm) × width (mm)^2^. When the tumor size reached approximately 100 mm^3^, the mice were randomly divided into two groups (six mice per group). TFP or vehicle were administered once daily (40 mg/kg) *via* intraperitoneal injection (i.p.).

### Analysis of PD-L1 and PD-1 Expression *In Vitro* in CRC Cells and in the Tumor Tissues

After treatment with TFP for 48 h, human HCT116 and mouse CT26 CRC cells were stained with antibody against PD-L1 and then analyzed by FCM. At the end of treatment of mouse CT26 subcutaneous models in immune-competent female BALB/c mice, we obtained single cell suspensions from the subcutaneous tumor tissues by mechanical and enzymatic dispersion as described previously (Belle et al., 2016). Then, one million cells were suspended in PBS and stained with antibodies against mouse CD4, CD8, CD45, PD1, and PD-L1. The cells were then analyzed by FCM.

### Immunohistochemical Staining of Tumor Sections

At the end of treatment of human HCT116 xenograft models, tumor tissues from the mice were collected and fixed in 4% paraformaldehyde. Then, the expression of cleaved caspase-3 and Ki-67 in the tumor tissues was detected as described by us and others using a DAB Detection Kit (Xia et al., 2014b; Ranjan et al., 2015).

### Statistical Analysis

The data are expressed as the mean ± the standard error of the mean from independent replicates and analyzed by GraphPad Prism 5. The differences between two groups were analyzed using two-tailed Student’s t-tests. P < 0.05 was considered statistically significant.

## Results

### Inhibitory Effects on TFP on CRC Cells *In* *Vitro*

**Figure 1:**
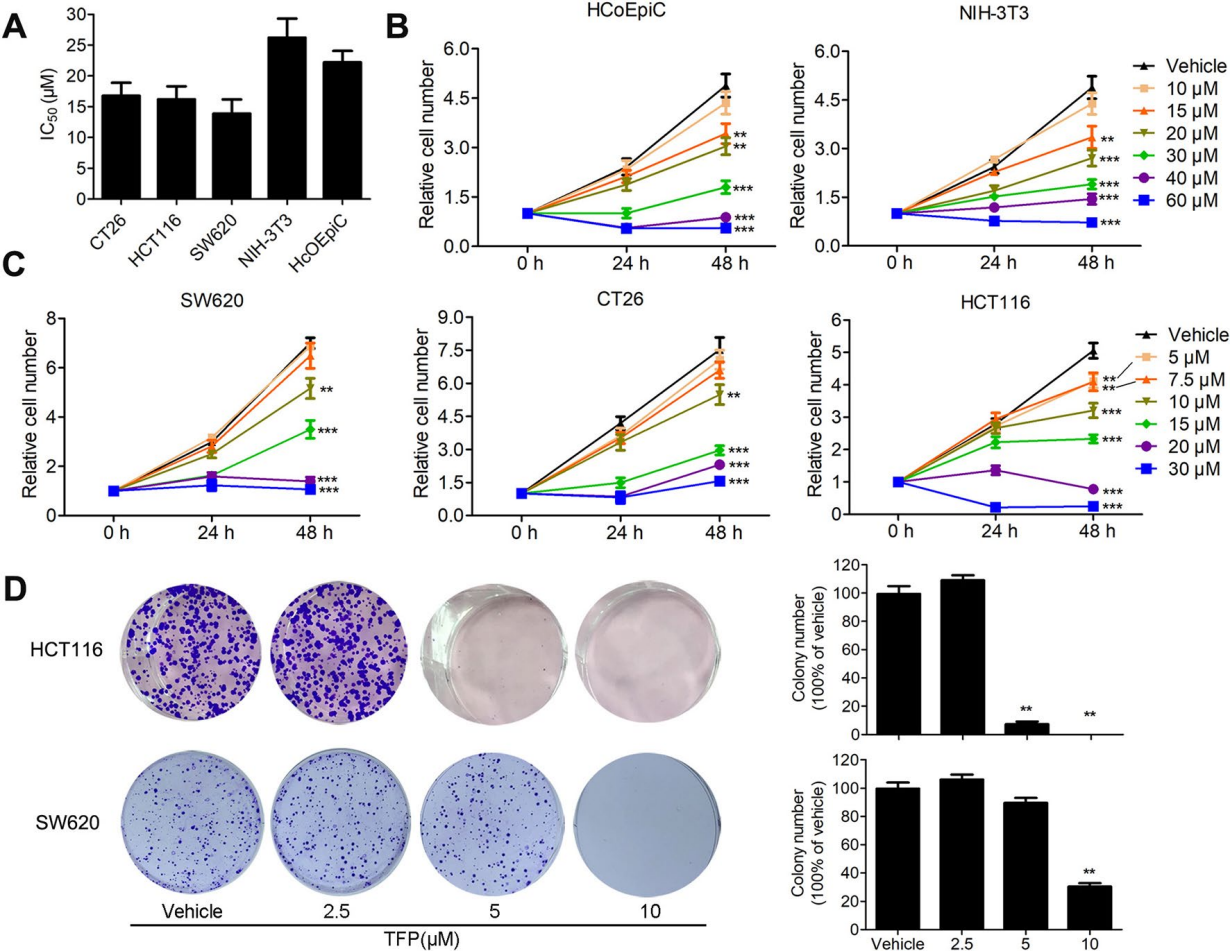
The inhibitory effects of TFP on the viabilities of CRC cells and non-tumorigenetic cells. **(A)** Inhibitory effects of TFP on three CRC cell lines and two non-tumorigenetic cell lines after 48 h of treatment (IC_50_ values, µM). **(B and C)** Cell proliferation curves of two non-tumorigenetic cell lines **(B)** and three CRC cell lines **(C)** after treatment with TFP at a range of doses for 48 h. Cell viabilities at each time point were measured by MTT assay and normalized to that at 0 h. **(D)** Effects of TFP on colony formation in CRC cell lines. Quantification is shown to the right of each cell line. **P < 0.01, ***P < 0.001.

The inhibitory effects of TFP against some CRC cell lines have been evaluated in our lab previously (Feng et al., 2018). Our current data showed that TFP displayed considerable antiviability activities against SW620, HCT116 and CT26 cells with IC_50_ values of 13.9, 16.2, and 16.8 µM, respectively, after 48 h of treatment (**Figure 1A**). We also got the proliferation curves of the cells during TFP treatment (**Figure 1C**). As we can see, 20 µM TFP decreased viable cell numbers in HCT116 and SW620 cells. The decreased cell numbers implied cell death after TFP treatment. However, CT26 cell numbers increased even after 30 µM TFP treatment although it showed inhibitory effects at this concentration. We did colony-forming assays to further investigate TFP’s activities on CRC cell growth. The plating efficiency of HCT116 and SW620 cells are approximately 39.8% and 30.8% when calculating the number of colonies, respectively. The results confirmed that TFP strongly inhibited the colony formation of CRC cells (**Figure 1D**). Meanwhile, TFP showed less inhibition on the viabilities of two non-tumorigenetic cell lines (**Figures 1A, B**). Collectively, the above data demonstrated that TFP could substantially inhibit CRC cell viabilities *in vitro*, and it showed some anti-viability selectivity on CRC cells *versus* non-tumorigenetic cells.

### TFP Synergized With 5-Fluorouracil and Oxaliplatin in CRC *In* *Vitro*

**Figure 2:**
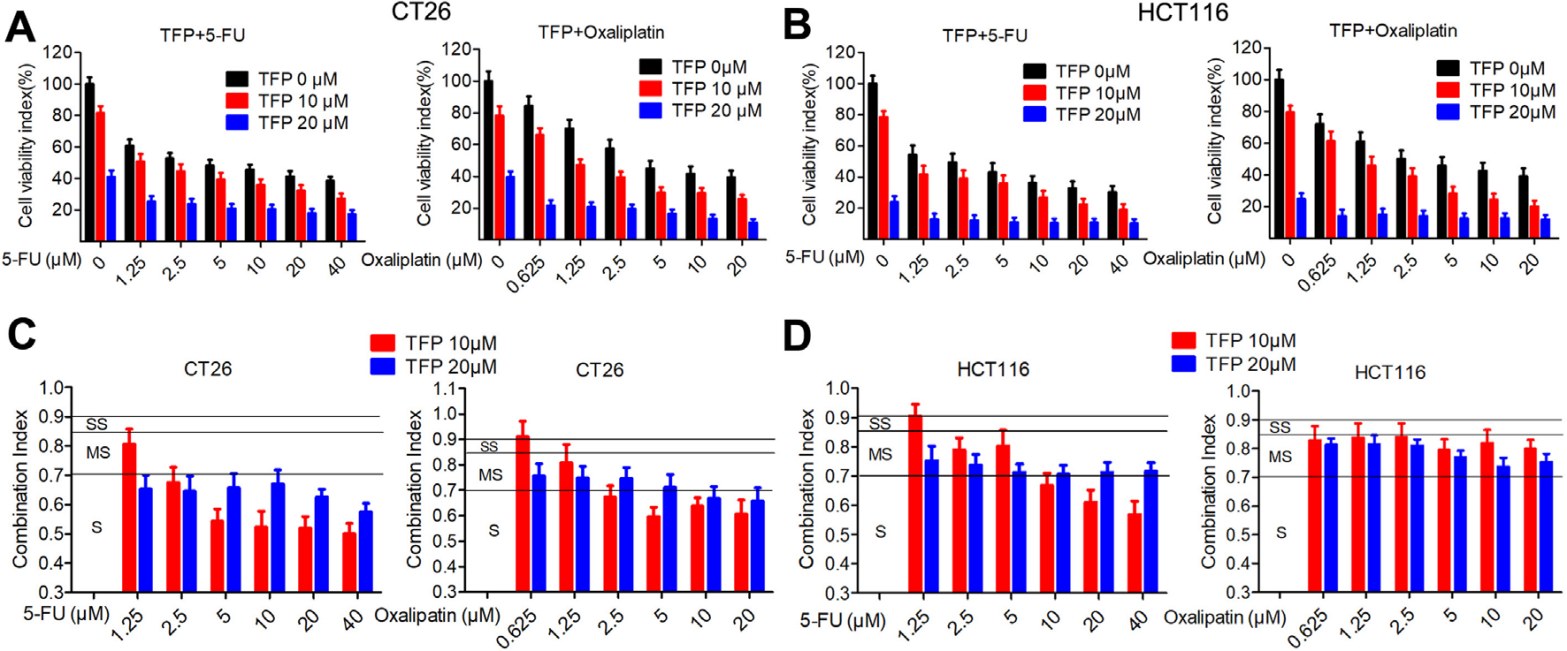
TFP synergizes with 5-fluorouracil and oxaliplatin in CRC *in vitro*. **(A and B)** Dose response for TFP and its combination with 5-FU and oxaliplatin in different ratios in CT26 cells **(A)** and HCT116 cells **(B)** after treatment for 48 h. Cell viabilities were measured by MTT assay. The cell viabilities of vehicle-treated cells were counted as 100%. **(C and D)** Combination index (CI) values for TFP and two standard chemotherapy drugs. CI values were calculated using the CompuSyn software based on the inhibition rate at each combination for CT26 cells **(C)** and HCT116 cells **(D)**. Slight synergism (SS, CI is between 0.85 and 0.9), moderate synergism (MS, CI is between 0.7 and 0.85), and synergism (S, CI is between 0.3 and 0.7).

5-Fluorouracil (5-FU) and oxaliplatin are commonly used chemotherapy drugs to treat CRC in the clinic. The IC_50_ values of TFP against the CRC cells are relatively high. Therefore, we investigated the combination effects of TFP and standard chemotherapy drugs *in vitro*. The data showed that TFP synergized with 5-FU and oxaliplatin to inhibit the viabilities of CT26 and HCT116 cells (**Figures 2A, B**). To quantify the effects of the combination, we calculated the CI using CompuSyn software. Most CI values are less than 0.85, indicating most combinations showed at least moderate synergism (**Figures 2C, D**). Clearly, the synergism effects are stronger in CT26 cells than that in HCT116 cells. Our data showed that TFP might synergize with the two chemotherapy drugs if we design appropriate drug combinations (Chou, 2006).

### TFP Induced G0/G1 Arrest in CRC Cells *In Vitro*

**Figure 3:**
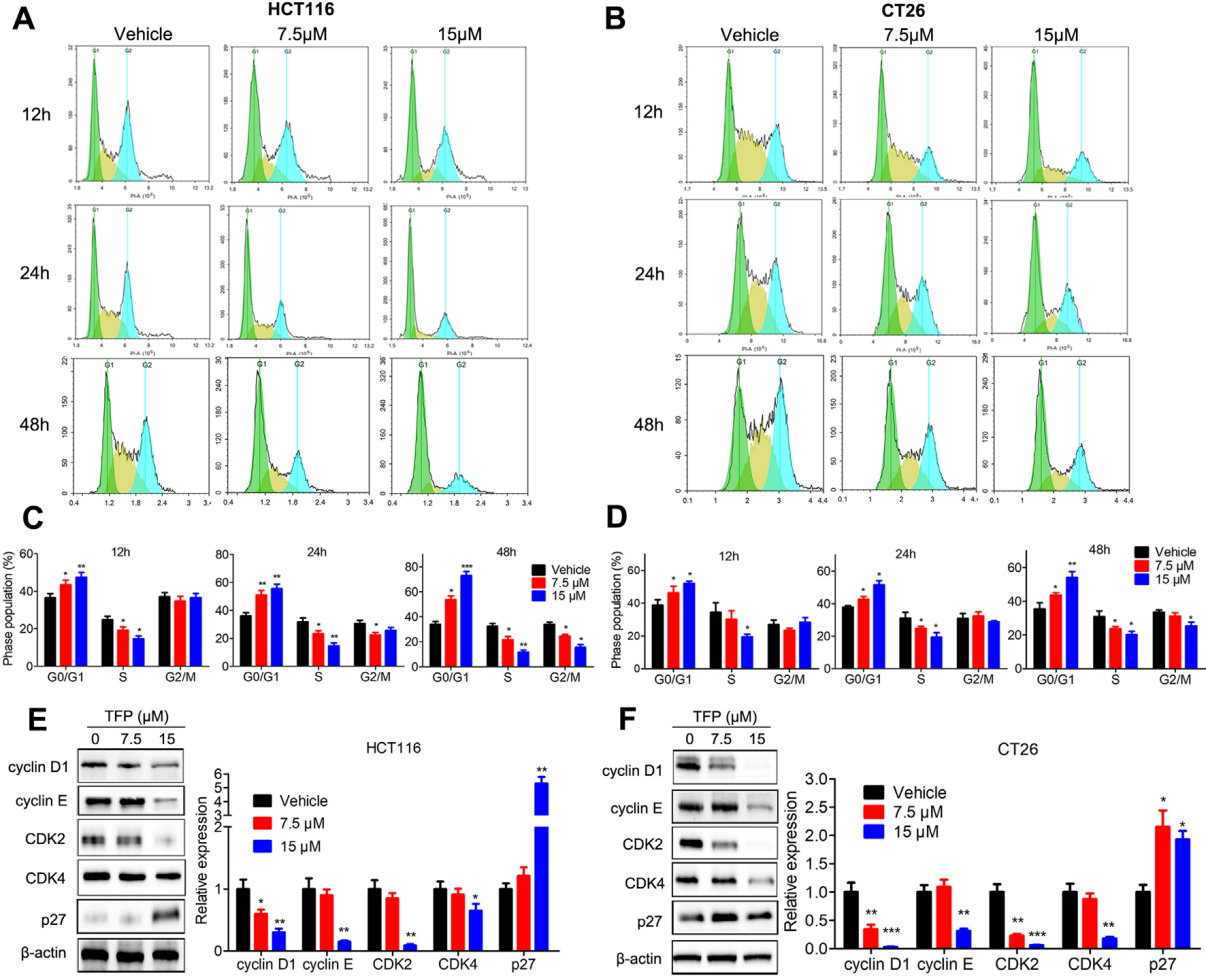
TFP induced G0/G1 arrest in HCT116 and CT26 cells. **(A and B)** HCT116 **(A)** and CT26 **(B)** cells were exposed to TFP for 12, 24, or 48 h and then stained with PI. Cell cycle distributions were analyzed by FCM. **(C and D)** Quantification of cell cycle distribution after TFP treatment in HCT116 **(C)** and CT26 **(D)** cells. **(E and F)** Expression levels of proteins involved in G0/G1 regulation were determined by western blot in HCT116 **(E)** and CT26 **(F)** cells after 48 h of TFP treatment. Quantification of the protein expression levels is shown to the right of each cell line. *P < 0.05, **P < 0.01, ***P < 0.001.

Cell cycle analysis showed the significant accumulation of HCT116 and CT26 cells in the G0/G1 phase after TFP treatment for 12, 24, and 48 h (**Figures 3A–D**). Protein expression levels of G0/G1 phase–related proteins were also evaluated. The data showed that the expression levels of CDK2, CDK4, cyclin D1, and cyclin E were decreased, and the levels of P27 were increased in both cell lines after TFP treatment for 48 h (**Figures 3E, F**). The above data showed that TFP-induced cell cycle arrest at the G0/G1 phase contributes to the suppression of cell viabilities.

### TFP Induced Apoptosis in CRC Cells

**Figure 4:**
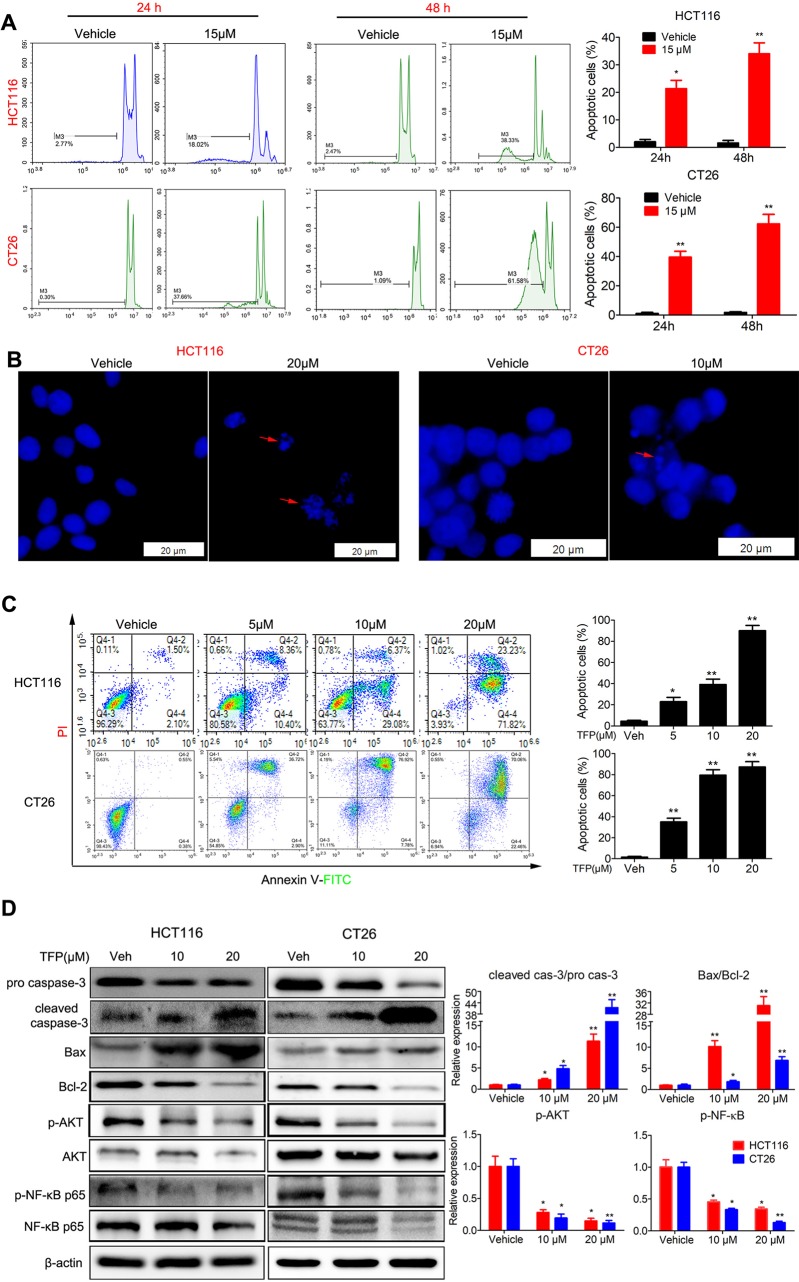
Effects of TFP on apoptosis in HCT116 and CT26 cells. **(A)** TFP treatment induced accumulation of sub-G1 cells in HCT116 and CT26 cells. The cells were treated with 15 µM TFP for 24 or 48 h and then stained with PI after fixing with 75% ethanol. The proportion of sub-G1 cells in each group was analyzed with FCM. **(B)** Fluorescence microscopy analysis of Hoechst 33342-stained HCT116 and CT26 cells after incubation with TFP for 24 h. The arrows indicate nuclear fragmentation. The scale bars represent 20 µm for both cell lines. **(C)** FCM analysis of cells stained with Annexin V-FITC/PI after treatment with TFP for 48 h. Quantification values are shown to the right of each cell line. **(D)** Expression levels of critical apoptosis-related proteins after TFP treatment for 48 h. Quantification of the expression levels is shown on the right. *P < 0.05, **P < 0.01.

**Figure 5:**
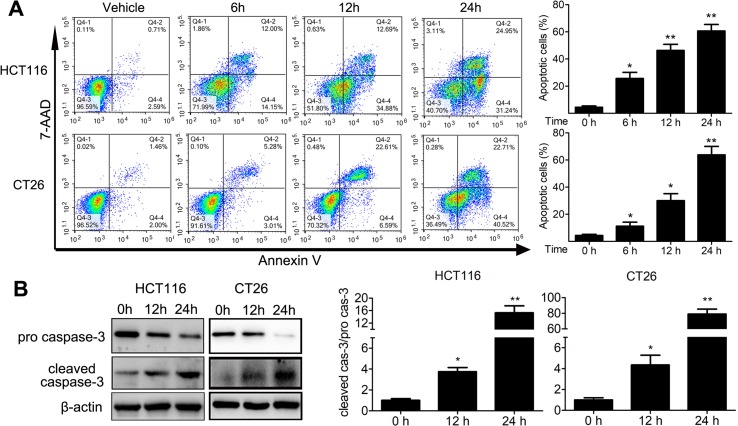
Time-dependent effects of TFP on apoptosis in HCT116 and CT26 cells. **(A)** The cells were treated with 30 µM (HCT116) or 20 µM (CT26) TFP for the indicated time. The apoptosis were analyzed by FCM after Annexin V/7-AAD labeling. **(B)** After treatment for 12 and 24 h, expression levels of pro-caspase-3 and cleaved caspase-3 were detected in HCT116 (30 µM TFP treatment) and CT26 (20 µM TFP treatment) cells. Quantification of the results is shown on the right. *P < 0.05, **P < 0.01.

We analyzed the proportion of sub-G1 cells when doing the cell cycle analysis. Interestingly, we found that TFP treatment led to accumulation of sub-G1 cells in both human HCT116 and mouse CT26 cells, indicating that TFP might induce apoptosis of CRC cells (**Figure 4A**). Then, we investigated the effects of TFP on apoptosis *via* many ways. Hoechst 33342 staining showed nuclear fragmentation, a characteristic of apoptosis, in HCT116 and CT26 cells after 24 h of TFP treatment (**Figure 4B**). Moreover, FCM analysis after Annexin V/PI staining quantitatively confirmed that TFP induced apoptosis in CRC cell concentration dependently after 48 h of treatment (**Figure 4C**). Evaluation of the expression levels of key proteins in apoptosis showed increased cleavage of caspase-3 (**Figure 4D**). Abnormal activation of AKT by NF-kB could lead to increased cell survival (Jeong et al., 2005). Consistently, TFP decreased the expression levels of both phosphorylated AKT and phosphorylated NF-kB (**Figure 4D**). These findings revealed that the induction of apoptosis contributed to TFP’s inhibiting activity toward CRC. We also investigate the time-dependent effects of TFP on inducing apoptosis of CRC cells. The data showed that TFP could even induce apoptosis after 6 h of treatment in both HCT116 and CT26 cells (**Figure 5A**). And, the apoptosis rate is rising with the treatment time. Consistently, TFP significantly increased the ratio of cleaved caspase-3/pro-caspase3 as early as 12 h after the treatment (**Figure 5B**).

### TFP Likely Induced Apoptosis *via the* Mitochondria-Mediated Intrinsic Apoptosis Pathway

**Figure 6:**
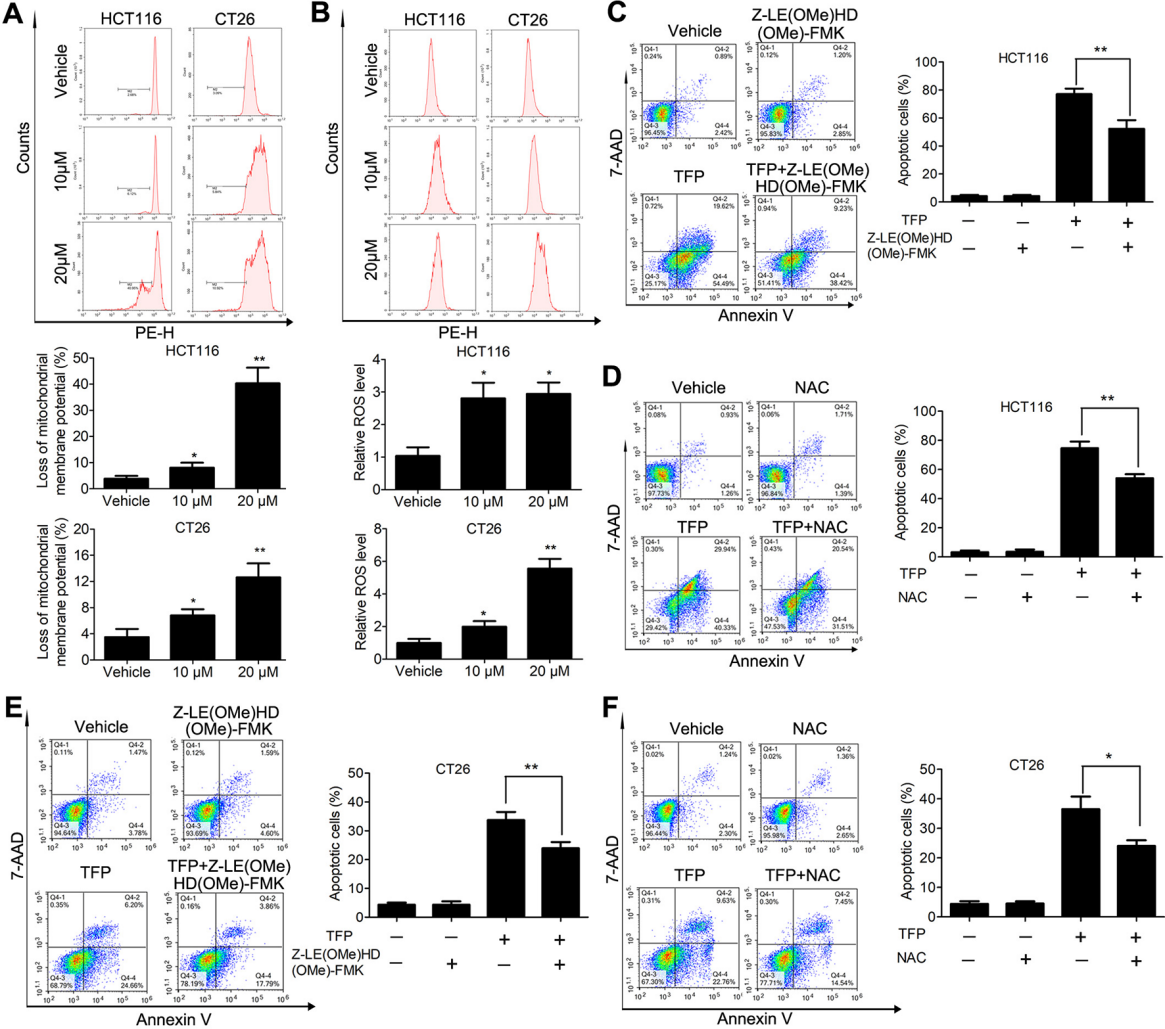
Effects of TFP on the mitochondria-mediated intrinsic apoptosis pathway. **(A)** TFP decreased ΔΨm of HCT116 and CT26 cells. The cells were treated with TFP (10 or 20 µM) for 24 h and then incubated with Rh123 to detect the change of ΔΨm by FCM. Quantified changes of ΔΨm based on three independent experiments were shown below the images. **(B)** TFP treatment elevated ROS levels in HCT116 and CT26 cells. After treatment with TFP (10 or 20 µM) for 24 h, the cells were stained with 10 µM DCFH-DA. The cellular ROS levels were detected by FCM. Quantified ROS levels based on three independent experiments were shown below the images. **(C)** HCT116 cells were treated with 20 µM TFP alone or in combination with Z-LE(OMe)HD(OMe)-FMK (caspase-9 inhibitor) for 48 h. Then, the apoptosis of the cells was measured by FCM after Annexin V-PE and 7-AAD labeling. Quantified values are shown to the right. **(D)** HCT116 cells were treated with 20 µM TFP alone or in combination with NAC for 48 h. Then, the apoptosis of the cells was measured. **(E)** CT26 cells were treated with 15 µM TFP alone or in combination with Z-LE(OMe)HD(OMe)-FMK for 24 h. Then, the apoptosis of the cells was measured. **(F)** CT26 cells were treated with 7.5 µM TFP alone or in combination with NAC for 48 h. Then, the apoptosis of the cells was measured. *P < 0.05, **P < 0.01.

Bcl-2 family proteins are critical for regulating mitochondrial integrity because they maintain the balance between anti-apoptotic and pro-apoptotic proteins (Cory et al., 2003). We analyzed the expression of some Bcl-2 family proteins in CRC cells after TFP treatment for 48 h. The results indicated that TFP decreased the expression of Bcl-2 significantly and increase the expression of Bax (**Figure 4D**). These findings implied that TFP might induce mitochondria-mediated intrinsic apoptosis. Loss of ΔΨm and increased ROS levels are associated with intrinsic apoptosis (Simon et al., 2000; Circu and Aw, 2010). As shown in **Figure 6A**, 24 h of TFP treatment led to a concentration-dependent loss of ΔΨm in both cell lines. Moreover, the ROS levels in both cell lines increased significantly after TFP treatment for 24 h (**Figure 6B**).

We also used a caspase 9 inhibitor (Z-LE[OMe]HD[OMe]-FMK) and the antioxidant NAC to evaluate which type of apoptosis is predominant in TFP-induced apoptosis in CRC cells. Obviously, our data showed that the caspase-9 inhibitor we used partially reversed TFP-induced apoptosis (**Figures 6C, E**). Meanwhile, the antioxidant NAC partially rescued the extent of TFP-induced apoptosis (**Figures 6D, F**).These data suggested that the mitochondria-mediated intrinsic pathway plays an important role in TFP-induced apoptosis.

### *In Vivo* Antitumor Activity of TFP and Mechanisms of Action

**Figure 7:**
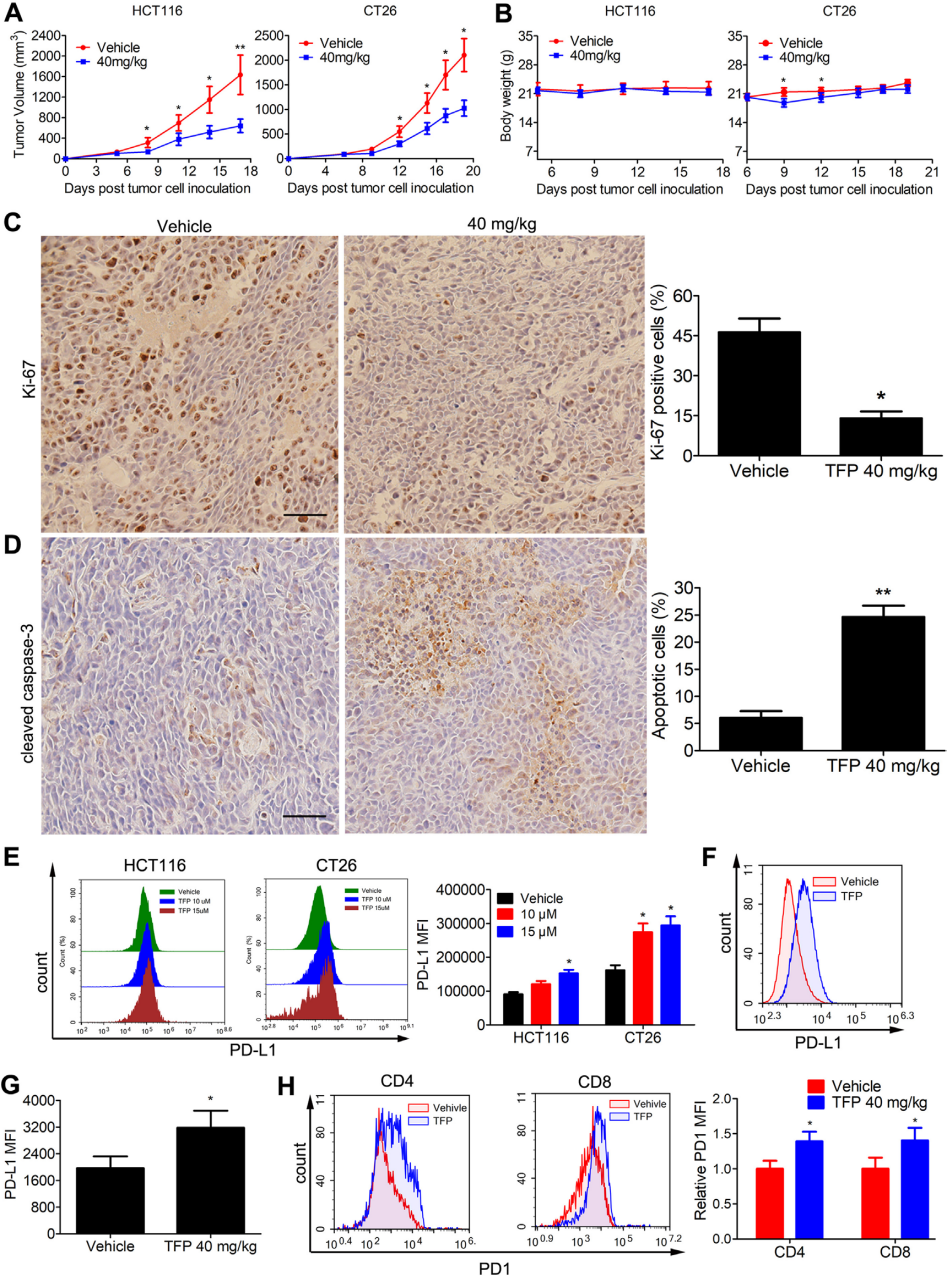
Effects of TFP on the growth of CT26 and HCT116 subcutaneous tumor *in vivo*. **(A)** Tumor volume changes in mice during TFP treatment. **(B)** Body weight changes of the mice during TFP treatment. **(C and D)** At the end of treatment of human HCT116 xenograft models in BALB/c nude mice, cleaved caspase-3 and Ki-67 expression levels in the tumor tissues were detected by Immunohistochemistry analysis. Quantification of the IHC analysis is shown to the right. **(E)** TFP treatment induced the expression of PD-L1 *in vitro* in cultural CRC cells. HCT116 and CT26 cells were treated with TFP for 48 h. The cells were stained with antibody against PD-L1 and analyzed by FCM. **(F–H)** Single cell suspensions were obtained from CT26 subcutaneous tumor tissues in immune-competent BALB/c mice at the end of treatment. The cells were stained with antibodies against mouse CD4, CD8, CD45, PD1, and PD-L1 followed by FCM analysis. **(F and G)** TFP treatment increased PD-L1 expression in the tumor cells from CT26 tumor tissues. Tumor cells were gated from CD45^−^ cells when performing the FCM analysis. **(H)** TFP treatment increased PD1 expression in tumor-infiltrating CD4^+^ and CD8^+^ T lymphocytes from CT26 tumor tissues. *P < 0.05, **P < 0.01.

Subcutaneous tumor models of human HCT116 and mouse CT26 cells were established to assess the in vivo anticancer abilities of TFP. Notably, TFP moderately suppressed tumor growth in both models without causing significant body weight loss (**Figures 7A, B**). The growth inhibition rates at day 17 and day 19 post-inoculation were 58.4 and 54% in HCT116 and CT26 models. Consistent with the in vitro data, TFP treatment suppressed the proliferation of tumor cells in the HCT116 tumor tissues from BALB/c nude mice as indicated by Ki-67 staining (**Figure 7C**). Furthermore, the considerable cleaved caspase-3 staining intensity indicated TFP-induced apoptosis in the tumor tissues (**Figure 7D**).

### TFP Modulated the Microenvironment in the Mouse Tumor Tissue

The PD-1/PD-L1 interaction represents an important immunosuppressive signaling pathway that facilitates tumor growth. We were curious about their role in the anticancer effects of TFP. Interestingly, TFP induced the expression of PD-L1 *in vitro* in both HCT116 and CT26 CRC cells (**Figure 7E**). We also measured PD-L1 expression of CT26 cells from the subcutaneous models at the end of the treatment. Notably, PD-L1 expressions in CT26 tumor cells were increased after TFP treatment (**Figures 7F, G**). Amazingly, TFP treatment induced PD-1 expression in the tumor-infiltrating CD4^+^ and CD8^+^ T cells from CT26 subcutaneous models (**Figure 7H**).

## Discussion

Despite tremendous efforts in the exploration of medical care, the clinical prognosis of CRC remains unsatisfactory (Wang and Li, 2012). More effective treatment strategies to improve the prognosis of patients with CRC are urgently needed (Wang and Li, 2012). The strategy of drug repurposing has been highly successful and provides more options for obtaining effective and inexpensive therapies from existing drugs (Gupta et al., 2013; Basso et al., 2018). In the present study, we adopted this strategy to explore the efficacy and mechanism of TFP, an FDA-approved antipsychotic drug, to treat CRC.

TFP is a promising anticancer candidate which has been extensively investigated. It has been shown to suppress several types of cancer, including breast cancer, lung cancer, hepatocellular carcinoma, and glioblastoma (Chi-Tai et al., 2012; Park et al., 2016; Jiang et al., 2017; Kang et al., 2017). Drug resistance and metastasis to other organs of the body are the two major obstacles to successful cancer treatment (Cao et al., 2018). TFP showed synergism with EGFR inhibitor and chemotherapy drug to overcome drug resistance in lung cancer by inhibiting cancer initiating cells (Chi-Tai et al., 2012). Most cancer patients died from metastasis. TFP exhibited anti-metastasis efficacies in many preclinical studies. Brain is a common metastasis site of lung cancer, breast cancer, and melanoma, and brain metastasis is a big threat to the patients’ survival and life qualities (Xu et al., 2019). Our previous work showed TFP suppressed brain metastasis and extended the survival of brain metastasis bearing mice in breast cancer models by inducing apoptosis and cell cycle arrest (Feng et al., 2018). The bone is a common site of metastasis from prostate, breast, and lung cancers. TFP also showed potentials to treat bone metastasis of breast cancer by antagonizing dopamine receptor D2 (DRD2) and suppressing bone resorption (Liu et al., 2018). In addition, another study showed TFP was able to reduce the angiogenesis and invasion of aggressive cancer cells *via* DRD2 to modulate the β-catenin pathway (Ashleigh et al., 2015). Thus, TFP might be a potential anticancer candidate.

However, its potential for CRC treatment remains unclear. In the present study, we found that TFP time-dependently suppressed the viabilities of several CRC cells. In addition, we demonstrated that TFP induced G0/G1 cell cycle arrest and mitochondria-mediated intrinsic apoptosis in CRC cells, and we proposed these as potential mechanisms mediating TFP’s suppressive effects on CRC.

TFP has been shown to induce G0/G1 cell cycle arrest in several studies (Jiang et al., 2017; Wang et al., 2017; Feng et al., 2018). An uncontrolled, abnormal cell cycle is one hallmark of cancer (Hanahan and Weinberg, 2011), and many proteins are involved in cell cycle regulation. Among them, many cyclin-CDK complexes are important regulators (Vermeulen et al., 2003). P27 is a strong CDK suppressor that physically interacts with cyclins, CDK2, and CDK4, to regulate the cell cycle at the G1/S transition (Coqueret, 2003; Besson et al., 2008). The current study showed that TFP treatment increased the expression of p27 and decreased that of cyclin D1, cyclin E, CDK2, and CDK4, implying that TFP induced G0/G1 arrest by regulating p27 and by disrupting the relevant cyclin-CDK complexes. The protein p27 is a well known CDK inhibitor that controls the cell cycle progression from G1 to the S phase upon mitogenic stimuli, and its increased expression would led to G0/G1 cell cycle arrest (Abukhdeir and Park, 2008). P27 can be regulated by several different independent mechanisms. FOXO3 is a key transcription factor that controls the transcription of numerous genes crucial for controlling cell cycle progression (Park et al., 2016). TFP has been shown to upregulate the expression of p27 through promoting forkhead box protein O3 (FOXO3) nuclear localization and activation.

TFP treatment induced apoptosis of cancer cells in lots of studies (Park et al., 2016; Jiang et al., 2017; Feng et al., 2018; Goyette et al., 2019). Deregulation of cell death is involved in the pathogenesis of cancer (Qi et al., 2018). There are three classical types of cell death processes—apoptosis, autophagy, and necrosis. They display distinct morphological characteristics by activating specific signaling cascades (Tang et al., 2019). During autophagy, autophagosome, a bilayer vesicle containing cytosolic materials and damaged organelles, is formed. Autophagosomes fuse with lysosomes to degrade cytoplasmic components, producing regenerative energy and metabolites for other cells. Autophagy promotes cell survival in most cases but can also induce autophagy-dependent cell death in specific circumstances, which is an active area in cell death research recently (Qi et al., 2018; Tang et al., 2019). Necrosis has long been recognized as an uncontrolled accidental cell death characterized by loss of membrane integrity and swelling of subcellular organelles (Tang et al., 2019). It is caused by a non-specific or non-physiological stress inducer and induces an inflammatory response caused by the release of intracellular components (Kono and Rock, 2008; Qi et al., 2018). Apoptosis, a caspase-mediated programmed cell death, is characterized by cell shrinkage, plasma membrane blebbing, exposure of phosphatidyl-l-serine (PS) on the outer plasma membrane (early stage), chromosome condensation, apoptotic body formation, and DNA fragmentation (late stage) (Fesik, 2005; Qi et al., 2018). According to their survival superiority, necrosis has the lowest superiority, followed by apoptosis, with autophagy that has the highest superiority. In addition to their independence, recent research advances indicate that these three types of cell death are interconnected with overlapping signaling cascades and crosstalk when exposed to different stimuli (Qi et al., 2018; Tang et al., 2019).

Dysregulation of apoptosis is associated with numerous pathological processes, including tumorigenesis (Tang et al., 2019). Thus, promoting apoptosis is a promising strategy to treat cancer. Apoptosis can be activated *via* either the extrinsic pathway or the mitochondria-mediated intrinsic pathway. Extrinsic apoptosis is activated upon the binding of death ligand to membrane receptors, especially death receptors, and is driven by caspase 8 and caspase 10 (Qi et al., 2018; Tang et al., 2019).The intrinsic apoptosis can be activated by numerous stress inducers such as DNA damage and oxidative stress. Maintenance of the mitochondrial membrane integrity and ΔΨm is essential for preventing intrinsic apoptosis. If the integrity is damaged, cytochrome c is released from the mitochondria into the cytoplasm to initiate apoptosis (Fesik, 2005; Xia et al., 2014a). During mitochondria-mediated intrinsic apoptosis, caspase 9 is activated and leads to the cleavage of caspase-3 and the death response (Xia et al., 2014a). Increased ROS levels are also involved in the intrinsic apoptosis process (Simon et al., 2000; Circu and Aw, 2010). We performed a FCM analysis and observed ΔΨm loss and increased cellular ROS levels in CRC cells. The Bcl-2 protein family play pivotal role in intrinsic pathway by modulating the permeability of the mitochondrial outer membrane (Qi et al., 2018). The pro-apoptotic protein Bax can open the ion channel in the outer mitochondrial membrane upon receiving a death signal, leading to mitochondrial membrane permeabilization and intrinsic apoptosis (Shimizu et al., 2000; Wei et al., 2001). In contrast, the anti-apoptotic Bcl-2 protein prevents cytochrome c release by suppressing the activities of pro-apoptotic proteins. Thus, the homeostasis of the Bcl-2 protein family is critical for cell survival, and disrupting this balance is a promising anticancer strategy (Cory et al., 2003). Our data strongly indicated that TFP treatment increased the Bax/Bcl-2 ratio. Meanwhile, we found that Z-LE(OMe)HD(OMe)-FMK, a caspase-9 inhibitor, partially rescued TFP-induced apoptosis. Moreover, the antioxidant NAC also partially reversed TFP-induced apoptosis. We investigated the apoptosis-inducing effects of TFP at several time points ranged from 6, 12, and 24 h and as long as 48 h. Our FCM and western blot data clearly showed TFP-induced apoptosis as early as 6 h, and this should not be a secondary cell death effects caused from other signaling pathway. Therefore, we propose that TFP may induce mitochondria-mediated intrinsic apoptosis in CRC cells, although other types of cell death may be associated with inhibition, and further research is needed.

NF-κB plays a pivotal role in CRC by promoting cell survival and resistance to apoptosis (Vaiopoulos et al., 2013). After nuclear translocation, it initiates transcription of genes involved in cell proliferation and survival, such as CDK2, cyclin D1, and Bcl-2 (Zubair and Frieri, 2013). Abnormal activation of AKT promotes cell survival and cell proliferation in many cancers (Roy et al., 2010), and NF-κB could upregulate the phosphoinositide 3-kinase (PI3K)/AKT cascade (Vaiopoulos et al., 2013). We found that TFP decreased phosphorylation of NF-κB and AKT, implying that inhibiting NF-κB/AKT signaling pathway contributed to TFP’s anti-CRC efficacy.

The tumor microenvironment has considerable effects on tumor growth and anticancer therapy response. Among the tumor microenvironment components, PD-L1/PD-1 axis plays an important role in dampening T cell activity against cancers (Sharma and Allison, 2015). Treatment with anti-PD-1 or anti-PD-L1 antibodies has shown long-lasting antitumor effects in patients with a variety of cancer types, especially in those exhibiting evidence of pre-existing PD-L1 expression (Hui et al., 2017). PD-1/PD-L1 blockade is a novel treatment option for CRC patients, especially those with DNA mismatch repair deficiency (Yaghoubi et al., 2019). Even for mismatch repair-competent CT26 cells, anti-PD-1 antibodies alone would cause tumor growth retardation in BALB/c mice (Singh et al., 2015). This demonstrated the potential of PD-1/PD-L1 blockade in CRC therapy. In oncology field, some small molecules drugs could influence the immune system by modulating the function and/or the proportion of immune cells *via* multiple mechanisms (Adams et al., 2015). They may enhance the anticancer immune effects. However, they may also have immunosuppressive effects and inhibit the effects of cancer immunotherapy. A recent study indicated that PARP inhibitors have a previously unknown activity to boost the immune response when used against tumor cells with weaknesses in repairing DNA (Chabanon et al., 2019). Although some studies suggested that HDAC inhibitors to be immunosuppressive and have negative effects on immune cell viability and function, increasing evidence also supported combining HDAC inhibitors with immunotherapy to obtain synergistic anticancer effects (Michiel et al., 2014). In TFP’s case, it had been shown to modulated immune system. It suppressed cytokine release from activated immune cells both *in vitro* and *in vivo* and improved survival rate in sepsis models (Park et al., 2019). Another study found that TFP combated respiratory and gastrointestinal bacterial pathogens probably through affecting the host immune system (Andersson et al., 2016). TFP have direct inhibitory suppressive effects on T cell proliferation, and the mechanism might be involved in PD-1/PD-L1 interaction (Roudebush et al., 1991). Thus, we were curious whether the PD-1/PD-L1 interaction was involved in the anticancer response of TFP in CRC. Here, we found that both PD-L1 expression in tumor cells and PD-1 expression in tumor-infiltrating CD4^+^ and CD8^+^ T cells were increased after TFP treatment in immune-competent mice. We hypothesized that the increased expression of PD-1 and PD-L1 might dampen the anticancer effects of TFP. The more PD-1/PD-L1 increases, the more effective the combination of TFP and immune checkpoint blockade. Although other components in the tumor microenvironment, including dendritic cells, macrophages, and exosomes, also express PD-L1 and might influence the total PD-L1 level in the tumor microenvironment (Johnson and Dong, 2017; Chen et al., 2018), our data suggest that immunological checkpoint inhibitors (such as anti-PD-L1 and anti-PD-1 antibodies) can be used with TFP to increase its effectiveness.

The direct anticancer target of TFP is still relatively unclear. Previous studies have shown that TFP might exert anticancer effects by modulating numerous signaling pathways. TFP exerts antipsychotic effects by suppressing central dopamine and serotonin receptor in patients (Park et al., 2019). Dopaminergic signaling has shown potentials to be a target for treating breast cancer tumor growth and bone metastasis (Liu et al., 2018). Indeed, TFP exhibited anticancer efficacies through antagonizing DRD2 in some studies (Ashleigh et al., 2015; Liu et al., 2018). TFP is also known to be a CaM modulator and prevents calcium (Ca^2+^) from binding to Ca^2+^-binding CaM, leading to increased cytosolic Ca^2+^ level (Park et al., 2019). Ca^2+^ signaling is a crucial signaling process involved in cell proliferation, and survival (Kang et al., 2017). It has been reported that TFP exerted anticancer effects by inhibiting the function of CaM in several cancer models (Yuan et al., 2015; Kang et al., 2017; Fancy et al., 2018). Other signaling pathways are involved in TFP’s anticancer effects. In lung cancer, TFP inhibited FOXO1 nuclear export and restored sensitivity to Erlotinib resistance by modulating the KLF6/FOXO1 signaling cascade in both cell culture and xenograft models (Sangodkar et al., 2012). Thus, TFP might be a potential anticancer candidate with multiple targets.

The drug dose we used in the animal studies is 40mg/kg/day, and the equivalent human dose should be 3.24 mg/kg/day when according to body surface area without changing the dosage form of the drug (Nair and Jacob, 2016). The dose for a person of 60 kg is 194.4 mg. The drug label information of TFP from the US NIH (https://dailymed.nlm.nih.gov/dailymed/drugInfo.cfm?setid=c2575a86-19e5-44df-8603-ff066bb9c9c5) states that most patients show optimum response on 15 mg or 20 mg daily, although a few may need 40 mg daily or more. So, the dose of 40 mg/kg in mice might be too high for clinical use. However, the current study is only a preliminary evaluation of TFP, and we can optimize its drug delivery to the tumor tissues and decrease drug uptake in normal tissues *via* numerous pharmacy optimizations (Bruschi, 2015). Therefore, we are able to decrease the drug dose and the resultant systemic toxicity, while improving the treatment effects. Meanwhile, the dose of 40 mg/kg is proved to be safe in our previous preclinical studies (Feng et al., 2018).

Here, we showed that TFP induced G0/G1 cell cycle arrest and mitochondria-mediated intrinsic apoptosis in CRC cells. However, the tumor microenvironment might dampen its anticancer abilities. In sum, our study signifies for the first time that the antipsychotic TFP is a novel treatment drug for CRC, and the combination treatment of TFP with immune checkpoint blockade might be used to increase antitumor efficiency.

## Data Availability

The raw data supporting the conclusions of this manuscript will be made available by the authors, without undue reservation, to any qualified researcher.

## Ethics Statement

The animal experiments were carried out in accordance with the recommendations of the Ethics Committee of Sichuan University. The protocol was approved by the Ethics Committee of Sichuan University.

## Author Contributions

YoX and CJ participated in most of the experiments. QX, JJ, and FX performed cell proliferation assay, cell cycle analysis, and animal studies. YaX, RW, and ZR performed apoptosis analysis and colony formation assay. FX and QX performed the IHC staining of tumor sections. YZ and TY conceived and supervised the project. YoX, CJ, YZ, and TY wrote the manuscript.

## Funding

This work was supported by the National Natural Science Foundation of China (81702898 and 81602950), China Postdoctoral Science Foundation (2018T110981 and 2017M612977), the Fundamental Research Funds for the Central Universities (2017SCU12046, the Postdoctoral Foundation of Sichuan University) and Post-Doctor Research Project, West China Hospital, Sichuan University (2019HXBH017).

## Conflict of Interest Statement

The authors declare that the research was conducted in the absence of any commercial or financial relationships that could be construed as a potential conflict of interest.
